# Differential effect of prenatal exposure to the Great Ethiopian Famine (1983–85) on the risk of adulthood hypertension based on sex: a historical cohort study

**DOI:** 10.1186/s12905-022-01815-w

**Published:** 2022-06-11

**Authors:** Kalkidan Hassen Abate, Getachew Arage, Habtamu Hassen, Jemal Abafita, Tefera Belachew

**Affiliations:** 1 Food and Nutrition Research Institute, Jimma University, Jimma, Ethiopia; 2 Department of Nutrition and Dietetics, College of Health Sciences, DebreTabor University, Debre Tabor, Ethiopia; 3 Department of Public Health, Hosanna College of Health Science, Hosanna, Ethiopia; 4 College of Business and Economics, Jimma University, Jimma, Ethiopia

**Keywords:** Ethiopian famine, Hypertension, Prenatal-exposure, Sex difference

## Abstract

**Background:**

The consequence of the Great Ethiopian Famine (1983–1985) on mortality had been well documented. However, the long term assaults of the famine on adulthood health, particularly on the risk of hypertension, has never been documented. The aim of this study was twofold: to examine the association of prenatal-exposure to the Great Ethiopian Famine and hypertension in adulthood and investigate if there existed sex difference in the risk estimate.

**Methods:**

Participants were recruited using multistage stratified random sampling and grouped as prenatal famine exposed and non-exposed cohorts based on their reported date of birth and current age. Independent sample T test was employed to compare continuous outcomes between the groups. A multivariable logistic regression was used to examine the association between prenatal famine exposure and risk of hypertension in adults.

**Results:**

Compared to the non-exposed groups, prenatal famine exposed cohorts had higher systolic blood pressure by 1.05 mmHg, (95% CI 0.29, 4.42) and diastolic by 2.47 mmHg (95% CI 1.01, 3.95). In multivariable logistic regression analysis, both unadjusted (COR = 2.50; 1.575, 3.989) and adjusted model for covariates (OR: 2.306 95% CI (1.426, 3.72) indicated a positive association between prenatal famine exposure and the risk of adult hypertension. However, in sex disaggregated analysis, the positive association was only significant in females (AOR = 3.95 95% CI 1.76, 8.85) indicating nearly four folds of odds of hypertension among females, while the corresponding figure for males was not significant (AOR = 1.201 (0.69, 2.07).

**Conclusions:**

Famine exposure during prenatal period could have differential impact on the development of hypertension based on sex, where adult exposed females had higher risk of hypertension as compared to males. Contextualized primary prevention programs with special focus on gender is critical undertaking in hunger spots and regions with historical famine.

**Supplementary Information:**

The online version contains supplementary material available at 10.1186/s12905-022-01815-w.

## Background

Cardiovascular diseases remain the leading cause of disease burden in the world and continues its rise exceptionally in low-income countries [1]. Globally, more than 1.4 billion of the world’s population suffers from hypertension [2]. According to the world health organization (WHO), the number of adults with hypertension increased from 594 million in 1975 to 1.13 billion in 2015, with an increase seen largely in low- and middle-income countries [3]. The rise in prevalence of hypertension and other chronic illness in low-income countries is becoming a growing concern for the health care system which is already overburdened by conventional infectious disease and undernutrition [1–4].

Knowledge on the impact of risk factors on hypertension including unhealthy diet, physical inactivity, consumption of tobacco and alcohol, overweight/obesity, family history of hypertension is well articulated in the past and supported by up-to-date evidence [3, 4]. Unfortunately, there has been marked increase in the distribution of these risk factors at par with the ongoing economic transitions in developing countries bringing a novel threat without precedent for their poor health care system [5, 6]. More importantly, the changes in dietary pattern (nutrition transition) which involve consumption of more palatable energy-dense diets containing snack foods, carbonated sweetened beverages, commercially available alcoholic beverages, accompanied by urban lifestyle is believed to be the major precursor of the rise in overweight/obese population, consequently resulting in higher risk of hypertension [3, 7].

Apart from the above established risk factors, the impact of the long-term effect of adverse prenatal environment on the development of hypertension during adulthood cannot be ignored, especially in settings known for past history of repeated famine and starvation [8–10]. As it was first illustrated by “The Barker hypothesis” (1990), an adverse nutrition in early life, including prenatally as measured by birth weight, increased susceptibility to obesity, diabetes, insulin insensitivity, hypertension, and hyperlipidemia and complications that include coronary heart disease and stroke [11]. In line with Barker, few studies documented higher proportion of hypertension among adults who had survived early life adversaries including famine [9, 12–20]. However, there still existed studies which reported null association between early-life famine exposure and adulthood hypertension warranting further investigation on the matter [21–23].

The Great Ethiopian Famine (1983 to 1985) is one of the worst disaster in living memory of the recent human history, which left 1.2 million dead, 2.5 million people internally displaced and nearly 200,000 children orphaned [24, 25]. Though the mortality and socio-economic impact of the famine have been well documented, the long term sequel of the assaults on adulthood health including the risk of hypertension has never been elucidated. Hence, primarily we set out to examine the association of prenatal famine exposure and risk of adulthood hypertension among the survivors of the great Ethiopian famine. In the meantime, we also aimed to investigate if there existed sex difference in risk of hypertension among the survived adults, given the importance of consideration of gender in public health interventions [5–7].

## Methods and materials

### Study setting, design and period

A historical cohort study was conducted in Raya Kobo District, Wollo, Ethiopia, to investigate the effect of early life famine exposure on risk of hypertension among adults. Raya Kobo District is found 408 km from Bahirdar the capital city of Amhara regional state. The district has the total population of more than of 228,798 of whom 147,837 (64.62%) are females [26].The district has 36 kebeles (the lowest local administrative units) of which 32 were rural. Raya Kobo is the epicentre of the 1983–1985 Ethiopian famine [24, 25, 27]. The study was conducted in the period of March 15 to April 30, 2019.

### Population and sampling procedures

Adults who were born between 1983 and 1985 were source population while the study population were those exposed to the Ethiopian great famine during their prenatal life. Those who were geographically displaced during the famine, physically disabled participants with deformity (Kyphosis, Scoliosis, and limb deformity), lactating and pregnant women were excluded. The sample size was calculated by applying two population proportion formula using Epi-Info version 7 and taking type one error 5%, 80% power, a design effect of 2, 5% non-response rate and a 1:1 ratio of exposed group to non-exposed group (r = 1). Assuming the proportion of hypertension in prenatal exposed group (27%) and non-exposed group (13%) from a study conducted in Biafran Famine study in Nigeria [19].

Multistage stratified random sampling technique was used to recruit our sample. North Wollo zone has 32 rural Districts and 4 town administrators. First, one third of Raya kobo District and Raya kobo town Kebeles was selected. Then baseline survey was conducted on selected Kebeles to identify eligible participants and a sampling frame was prepared. Finally, study participants were selected from each kebeles using simple random sampling method from the prepared registration book (Additional file 1).

### Famine exposure status

Study participants were categorized into prenatal famine exposed and non-exposed cohorts based on their birth dates and age. Accordingly, study participants who were born from August 8, 1983 to August 30, 1985 (aged 34–36 years) were classified as prenatal exposed cohorts. Study participants who were born from September 8, 1987 to October 8, 1988 (aged 30–32 years) were classified as the non-exposed cohorts. In order to reduce misclassification, a one-year transition time (washout time) was considered and hence, adults who were born between September 8, 1986 and August 30 1, 1987 were excluded (Additional file 2).

### Measurements and variables

The WHO steps instrument inform our entire standard operating procedures for data collection [28] The outcome of interest, hypertension, was determined by either participant report of current use of anti-hypertensive medication or through direct blood pressure (BP) measurement records where the diastolic blood pressure (DBP) ≥ 85 mmHg or systolic blood pressure (SBP) ≥ 130 mmHg in at least three measurements and confirmed in a follow-up visit or a previous diagnosis of hypertension by a clinician is considered hypertension [29]. Blood pressure was measured by trained professional nurses in triplicate using digital apparatus after 20 min of rest of participants contact. The subsequent measurements were done 5 min apart. During data analysis the average BP reading was taken for final analysis. Weight was measured and recorded to the nearest 100 g (g) using portable battery operated Seca® digital scale and height to the nearest 0.1 cm (cm) using a stadiometer (Seca®, Germany) with the participants positioned at the Frankfurt Plane.Weight and height were measured in triplicates; average values were taken. To minimize inter-observer error, standardization exercise was done and the scale was checked read zero and calibrating using an object of known weight before measurement.

### Assessment of covariates

Socio-demographic information, family and personal history of chronic illness, lifestyle factors including cigarette smoking, alcohol drinking, Khat chewing, physical activity, and diet pattern were collected during face-to-face interviews by trained interviewers. Household wealth status was assessed by asking about household assets and utilities were used finally to generate household wealth index [30]. Physical activity was assessed using the International Physical Activity Questionnaire (IPAQ) and categorized as low, medium and high [31]. Dietary pattern was assessed using qualitative food frequency questionnaire (FFQ) composed of 38 food items covering the main foods consumed in the study area [32, 33]. The food frequency questionnaire (FFQ) was pre-tested on 45 study participants and reliability was checked (Cronbach’s α coefficient = 0.80). Participants’ use of the specified substances (smoking, drinking and Khat chewing) in the past 3 months was considered as current use [34]. Family history of diabetes mellitus/ hypertension was assessed from the response of grandparents, mother/father and siblings only.

### Data management and analysis

The data were checked for inconsistencies and completeness before entered and then double entered using EpiData software version 3.1 and transported to SPSS software version 23 for analysis. Data were edited and cleaned by running a simple frequency, cross tabulations and sorting to check for inconsistencies, completeness and outliers. Data are presented as mean and standard deviation (± SD) for continuous variables, and proportion (%) for categorical variables.

Group differences between exposed and non-exposed cohort were tested using independent T-test for continuous variables with normal distribution and chi-square test for categorical variables. Principal component analysis was done to generate household wealth tertiles by asking about household assets and utilities. K-means cluster analysis was used to assess dietary pattern. Finally two major dietary patterns were identified as healthy and unhealthy dietary pattern based on the participant’s fruits and vegetable consumption.

Binary logistic regression analysis was used to obtain the crude and adjusted odds ratios (OR) with 95% confidence interval (CI) and for evaluation of association between prenatal famine exposure, possible confounder and hypertension. We also disaggregate the analyses by creating independent dummy variable (female exposed and male exposed) to evaluate the differential effect of famine exposure based on sex on risk of hypertension. All analyses were two-sided and p-value < 0.05 was considered as statistically significant.

## Results

### Study participants

A total of 700 study participants (350 prenatal famine exposed and 350 non-exposed) were included. Among the covariates Sex (p = 0.006), educational status (p = 0.001) and physical activity (p = 0.005) were different among the famine exposed and non-exposed groups. However, no significant differences were observed on body mass index, residence, and wealth tertiles, dietary consumption, smoking status, alcohol intake and Khat chewing among the two cohorts (Table 1).

**Table 1:** Basic characteristics of the study participants by famine exposure Status, in North Wollo Zone, Northeast Ethiopia, 2019 (n = 700)

Variables	Total N (%)	Prenatal- exposed n (%)	Non-exposed n (%)	P^a^
Sex				
Female	375 ( 53.6)	206 (54.9)	169 (45.1)	0.006
Male	325 (46.4)	144 (44.3)	181 (55.7)	
Residence				
Urban	123 (17.6)	70 (56.9)	53 (43.1)	0.112
Rural	577 (82.4)	280 (48.5)	297 (51.5)	
Educational status				
Cannot read and write	227 (32.4)	140 (61.7)	87 (38.3)	0.001
Primary school	152 (21.7)	86 (56.6)	66 (43.4)	
Secondary school	188 (26.9)	72 (38.3)	116 (61.7)	
Above secondary school	133 (19.0)	52 (39.1)	81 (60.9)	
Wealth tertiles				
Poor	232 (33.1)	124 (53.4)	108 (46.6)	0.438
Medium	193 (27.6)	93 (48.2)	100 (51.8)	
Rich	275 (39.3)	133 (48.4)	142 (51.6)	
Physical activity level				
Low	146 (20.9)	69 (47.3)	77 (52.7)	0.005
Moderate	328 ( 46.9)	148 (45.1)	180 (54.9)	
High	226 (32.3)	133 (58.8)	93 (41.2)	
Dietary consumption				
Unhealthy	605 (86.4)	297 (49.1)	308 (50.9)	0.27
Healthy	95 (13.6)	53 (55.8)	42 (44.2)	
Alcohol drinking				
Yes	466 (66.6)	227(48.7)	239(51.3)	0. 336
No	234(33.4)	123(52.6)	111(47.4)	
Khat chewing				
Yes	60 (8.6)	29 (48.3)	31 (51.7)	0.893
No	640 (91.4)	321 (50.2)	319 ( 49.8)	
Smoking				
Yes	19 (2.7)	8 (42.1)	11 (57.9)	0.642
No	681 (97.3)	342 (50.2)	339 (49.8)	

^a^Pearson’s chi-square test

The prevalence of hypertension among study participants in famine exposed and non-exposed group were 64 (18.3%) and 31 (8.9%), respectively. Independent T test indicate, compared to non-exposed groups, prenatal famine exposed cohorts had higher systolic blood pressure by 1.05 mmHg, (95% CI 0.29, 4.42, P = 0.025) and diastolic by 2.47 mmHg (95% CI 1.01, 3.95). In contrast, no difference in body mass index (BMI) was found between the groups (P = 0.514). (Table 2).

**Table 2:** Blood pressure and BMI by exposure to the Great Ethiopian Famine of 1983–1985 in North Wollo Zone, Northeast Ethiopia, 2019

Variables	Exposed (N = 350)	Non-exposed (N = 350)	Mean difference 95% CI	*p *value
SBP (mmHg),mean ± SD	116.25 ± 14.65	113.89 ± 13.09	1.05(0.29,4.42)	0.025
DBP (mmHg),mean ± SD	77.90 ± 10.07	75.42 ± 9.80	2.477(1.01,3.95)	0.001
BMI (kg/m^2)^	23.03 ± 4.15	22.65 ± 4.03		0.514
Hypertension n (%) (yes)	64(18.3)	31(8.9)		0.0004

SBP = systolic blood pressure; DBP = diastolic blood pressure; BMI = Body mass index P-value-represents tests of independent t-test for continuous variables or χ2-test for categorical variables

In the multivariable logistic regression analysis, all the three models: 1) the unadjusted model (Model 1: COR = 2.50; 1.57, 3.98), 2) adjusted model for covariates including age, residence, physical activity, dietary consumption, smoking status, alcohol consumption, Khat chewing and BMI (Model 2: AOR; 2.33; 1.44, 3.77) and the final model (Model 3: adjusted for covariates in Model 2, family history of hypertension and diabetes mellitus (AOR: 2.30 95% CI 1.42, 3.72) indicated a positive association between prenatal famine exposure and risk of hypertension during adulthood (column 1, Table 3). However, in all models, stratified by sex while adjusted for the same covariates, these positive associations were significant only in females (columns 2 and 3, Table 3). Accordingly, the final model indicate that famine exposed female adults had nearly four folds of odds of developing hypertension (AOR = 3.95 95% CI 1.76, 8.85) while males were not (AOR = 1.201; 0.69, 2.07) (Table 3).

**Table 3:** Association between famine exposure and hypertension in Raya Kobo District, North Wollo Zone, Northeast Ethiopia, 2019

	Exposed (N = 350)	Female exposed (N = 206)	Male exposed (N = 144)
Models	Odds ratio (95% CI)	Odds ratio (95% CI)	Odds ratio (95% CI)
Model 1	2.507 (1.575, 3.989)*	4.597 (2.085, 10.134)	0.965 (0.587,1.586)
Model 2	2.339 (1.449, 3.776)*	3.985 (1.780, 8.922)*	1.225 (0.711,2.109)
Model 3	2.306 (1.426, 3.729)*	3.953 (1.764, 8.857)*	1.201 (0.696,2.073)

Model 1; unadjusted. Model 2; adjusted for age, residence, physical activity, dietary consumption, smoking status, alcohol consumption, Khat chewing and BMI. Model 3; adjusted for residence, physical activity, dietary consumption, smoking status, alcohol consumption, Khat chewing, BMI, family history of hypertension and diabetes mellitus; * = significant

## Discussion

In the present study, we found that adults who had prenatal exposure to famine were 2.3 times more likely to have risk of hypertension as compared to non-exposed groups. In broader perspective, these findings were in line with the findings of the Biafran [19], Dutch [16] and Chinese famine studies [9, 12, 15, 17, 20, 35]. Earlier studies hypothesized possible mechanisms for such association of early life famine exposure and adult hypertension including metabolic adaptations, phenotypic plasticity, reduction in number of nephrons, alterations in metabolic pathways resulting in elevated oxidative stress, change in growth patterns due to reductive adaptation, gene expression and epigenetic modifications [36–40].

Contrary to the present study findings, the Leningrad Siege famine [23] and Dutch famine studies [21] reported that prenatal famine exposure was not associated with hypertension as compared to the non-exposed group. This could be partly explained by differences in definitions of famine exposure, geographical areas and characteristics of study participants. For example, both Dutch famine and the Leningrad Siege famine compared blood pressures of the exposed and non-exposed groups as continuous variable, not as hypertension. Furthermore, both studies involve older age participants where the physiological change associated with aging itself could hinder the independent effect of the exposure in both groups.

In the gender-disaggregated analysis, prenatal exposure to the Ethiopian famine was found to have significant association with elevated risk of hypertension in females, but not in males. This finding corroborates the results of a study in China [20], which reported fetal-infant and early childhood famine exposure were associated with an increased risk of hypertension in females, not in men. A similar observation has been documented by Liu et al. who reported that fetal-infant exposure to famine was associated with an increased risk of hypertension in females [35]. In addition, Shi et al. also found an increased risk of hypertension among rural females exposed to famine during utero or childhood life [8]. The observed sex difference can be explained by the phenomenon called “survivors bias”, meaning male infant were more vulnerable to immediate squeal of prenatal malnutrition than females and die at higher rates than females, affecting the characteristics of survivors [39, 40]. Consequently, these natural selection could lead to bias which affect estimates of health parameters in the adult population [41].

To our knowledge, our study is the first one to examine the association between famine and risk of hypertension in Ethiopia. We believe our finding is robust as it accounts possible covariates including residence, physical activity, dietary consumption, smoking status, alcohol consumption, Khat chewing, BMI, family history of hypertension and diabetes mellitus. However, the study could have few limitations; due to global nature of the famine we couldn’t locate true age matched non-exposed group in the same area. As no one survived the famine adversary, we take those adults in the same setting but younger in age by two years as control. Furthermore, the date of birth was self-reported, hence participant to recall bias. However, due to the damage incurred by the famine, it is unlikely to fade on living memory of the survivors’ story, thus it could not be a threat to the findings of the study.

## Conclusions

Famine exposure during prenatal period have differential effect on development of hypertension based on sex, where females do have higher risk of hypertension. Contextualized primary prevention programs with special focus on gender is critical in hunger spots of the region.

## Supplementary Information

**Additional file 1.** Sampling procedure.

**Additional file 2.** Window of exposure of the Ethiopian Great Famine.

## Data Availability

All data generated or analysed during this study are included in this published article [and its supplementary information files/additional file].

## Abbreviations

CI: Confidence interval
BMI: Body mass index
COR: Crude odds ratio
AOR: Adjusted odds ratio
PCA: Principal component analysis
FFQ: Food frequency questionnaire (FFQ)
SD: Standard deviation
