# Supplementary material for: Differential effect of prenatal exposure to the Great Ethiopian Famine (1983–85) on the risk of adulthood hypertension based on sex: a historical cohort study
Source: BMC Womens Health. 2022 Jun 11;22:220. doi: 10.1186/s12905-022-01815-w (PMC9188157; doi:10.1186/s12905-022-01815-w)
Supplement: Supplementary file 1 — Additional file 1. Sampling procedure. [file 12905_2022_1815_MOESM1_ESM.docx]

Registration was carried out to prepare sampling frame

Total participants (n = 997)

Proportional allocation to selected kebeles then participants were selected by simple random sampling methods

Exclusion and

Missing data (n= 297)

Participants included in the study (n= 700)

1. Prenatal exposed (n = 350)
2. Non exposed group **(**n = 350)

Supplementary figure 1. Flow diagram representing sample recruitment.
