# Supplementary material for: Differential effect of prenatal exposure to the Great Ethiopian Famine (1983–85) on the risk of adulthood hypertension based on sex: a historical cohort study
Source: BMC Womens Health. 2022 Jun 11;22:220. doi: 10.1186/s12905-022-01815-w (PMC9188157; doi:10.1186/s12905-022-01815-w)
Supplement: Supplementary file 2 — Additional file 2. Window of exposure of the Ethiopian Great Famine. [file 12905_2022_1815_MOESM2_ESM.docx]

Window of exposure to the 1983-1985 Ethiopian Great Famine cohorts, North Wollo Zone, 2019

| **Birth date of participants (dd/mm/yyy)** | **Exposure to the famine August 1983, August 1985** | **Age at exposure to the famine** | **Age during the recruitment in the present study** |
| --- | --- | --- | --- |
| 08/August/1983-30/August /1985 | In utero exposed | born or conceived during the famine | 34-36 |
| 08/September/1986 30/August/1987 | Transition (Washout period) | One years after the famine | 33 |
| 08/September/1987-08/October/1988 | unexposed (control group) | Two years old after the famine | 30-32 |
